# Fiji plugin for annotating movies with custom arrows

**DOI:** 10.1242/bio.056200

**Published:** 2020-12-01

**Authors:** Stephan Daetwyler, Carl D. Modes, Reto Fiolka

**Affiliations:** 1 Department of Cell Biology, UT Southwestern Medical Center, Dallas, 75390, Texas, USA; 2 Max Planck Institute for Molecular Cell Biology and Genetics, 01307 Dresden, Germany; 3 Center for Systems Biology Dresden, 01307 Dresden, Germany; 4 Cluster of Excellence Physics of Life, TU Dresden, 01069 Dresden, Germany; 5 Lyda Hill Department of Bioinformatics, UT Southwestern Medical Center, Dallas, 75390, Texas, USA

**Keywords:** Fiji plugin, Annotation, Arrow, Arrowhead, Movie, Time-lapse data

## Abstract

Annotation of time-lapse data provides an important tool to highlight dynamic processes. Particularly, arrows, circles and arrowheads are useful to pinpoint a specific process, stationary or evolving over time. Here, we describe a user-friendly Fiji plugin to facilitate annotation of movies with arrows, arrowheads and circles. The plugin also enables saving and loading of annotated tracks.

This article has an associated First Person interview with the first author of the paper.

## Abstract (precis)

**Summary:** We present a user-friendly Fiji plugin to annotate movies with custom arrows, thereby providing biologists with no coding experience an easy tool to highlight dynamic processes in their movies.

## INTRODUCTION

Effective scientific communication relies on highlighting and annotating relevant processes in images and movies. For this, circles, arrows and arrowheads are among the most used graphical symbols for scientific illustrations (Wong, 2011). They effectively guide us through complex data and images. Therefore, many software and visualization tools such as the Arrow Tool (Tinevez et al., 2017) in ImageJ/Fiji (Schindelin et al., 2012) have been designed to facilitate drawing these graphical symbols. While efforts have been put into making these tools applicable for annotating processes in movies (Carpentier, 2007; Straatman, 2018), the applicability and user-friendliness of these tools to highlight non-stationary processes is limited. Consequently, most scientists have been adding graphical symbols in a tedious frame by frame manner. Here, we present a novel, user-friendly Fiji plugin for annotating stationary and non-stationary processes with circles, arrows and arrowheads. Specifically, the plugin enables easy labeling of processes by clicking, and interpolation between frames to reduce the number of frames that have to be annotated. Moreover, the trajectory of the process of interest can be saved for further analysis and transferred to annotate another movie. This is particularly useful for applying the same annotation to several color channels.

## RESULTS

**Fig. 1.**
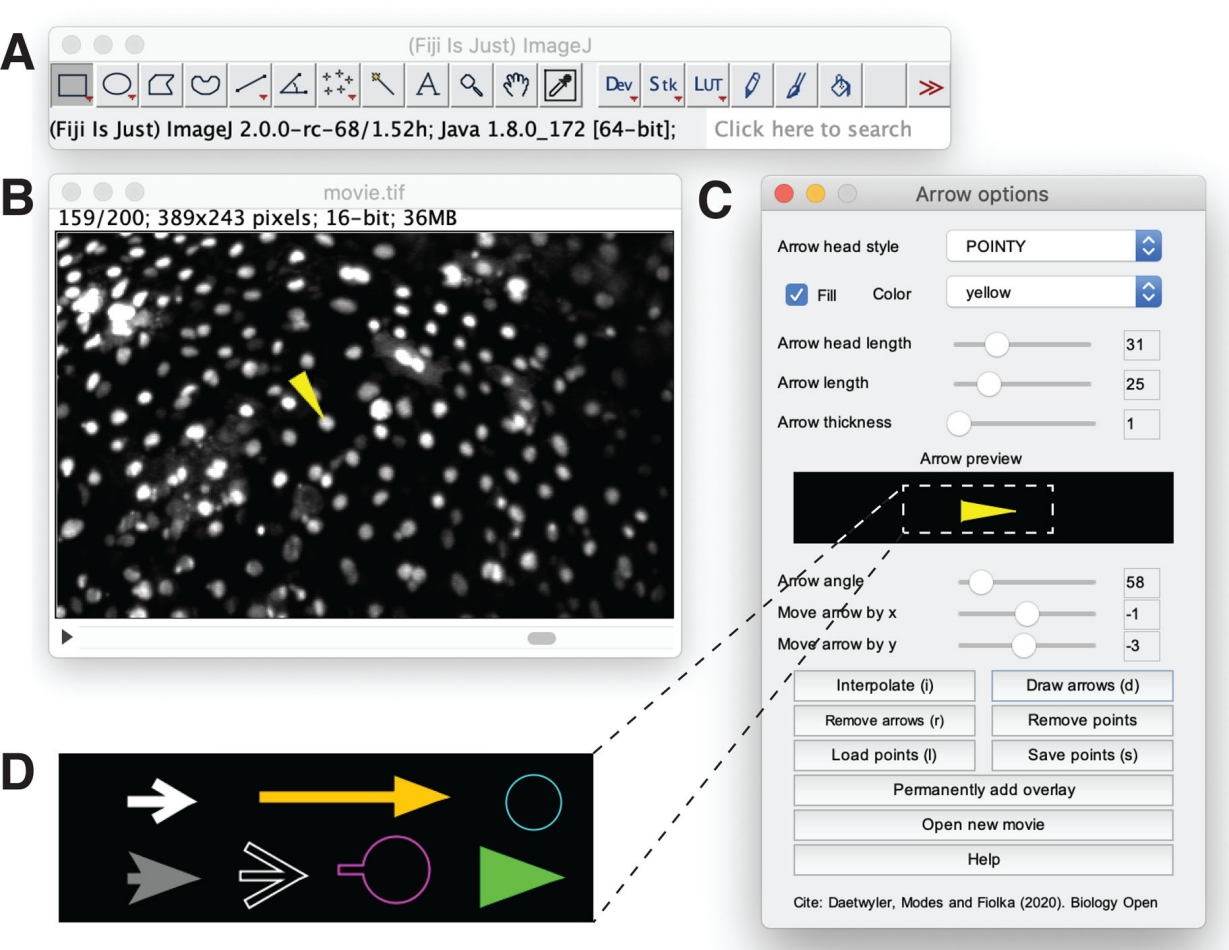
**Graphical user interface.** The graphical user interface to add custom graphical symbols to a movie is run in (A) Fiji. When the rectangular selection tool in the Fiji menu bar (A) is selected, the user can interact with the movie (B) to generate a trajectory of the process of interest. In the arrow options panel (C), the user chooses the style (shape), filling and color of the added graphical symbol. The current graphical symbol specified by the selected arrow head style, head length, length, thickness, fill status and color is previewed in an ‘Arrow preview’ field. The user can further determine the angle, and position (x and y) of the selected graphical symbol relative to the selected trajectory. Moreover, buttons on the arrow options panel help the user to navigate the annotation process and allow the user to open a new movie to annotate. (D) Selection of possible designs for the graphical symbol are obtained by changing the parameters in the arrow options panel.

We designed a user-friendly plugin for Fiji (Schindelin et al., 2012) to add custom graphical symbols such as arrows to movies (Fig. 1). We chose Fiji as platform as it is a widely used, freely available and open-source platform that runs on Windows, Mac and Linux. The plugin was implemented as ImageJ 1.x plugin in Java. An overview about the Java classes at play is given in the Materials and Methods section and the commented source code of the plugin is available at: https://github.com/DaetwylerStephan/draw_arrow_in_movies. Here, we describe how a user can use the plugin to easily annotate a movie. A tutorial video about how to use the plugin is also available (https://youtu.be/Yx82zZsPiYg).

### Installation

The plugin requires Fiji to run. Fiji is available at http://fiji.sc/. After installing Fiji, download the compiled plugin ‘SPIM_DrawArrowInMovie_-14.0.0.jar’ from github: https://github.com/DaetwylerStephan/draw_arrow_in_movies. Install it by drag and drop the .jar file into the Fiji toolbar or over ‘Plugin>Install PlugIn…’. After installation of the plugin, a restart of Fiji is required.

### Running the plugin

**Fig. 2.**
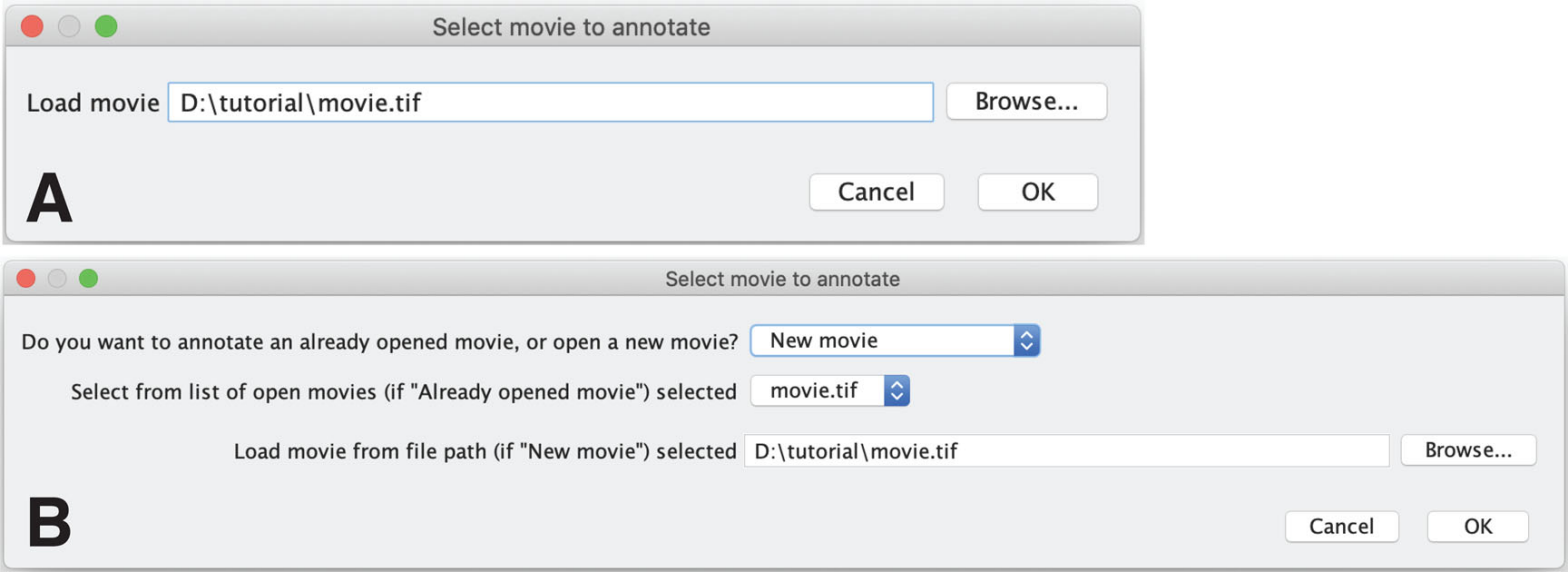
**Startup dialog window.** After starting the plugin or when opening a new movie from the Arrow options panel, a dialog window ‘Select movie to annotate’ opens. The dialog window is different based on whether a movie has already been opened in Fiji. (A) If no movie is open, the dialog window asks for a file path to a movie to annotate. This file path can either be specified as a single .tif file or as folder containing single frame .tif files. (B) If a movie is already open in Fiji, the user has the option to annotate this movie by selecting the option ‘Already opened movie’ in the first question and choosing the movie from the list of open images. Alternatively, a user can open a new movie from disk by selecting ‘New movie’ and loading it by specifying the file path as in A.

After installation, the plugin is found in the Plugin folder of Fiji. Navigating and clicking on the menu item ‘Plugins>Annotate_movie>Draw_Arrows’, starts the plugin and a startup dialog window opens (Fig. 2) to specify the movie to annotate. Please note that if you want to annotate a hyperstack of several channels over time, convert it first to an RGB stack.

If no movie is open in the current active Fiji session, the startup dialog window only asks for a file path in the field ‘Load movie’ (Fig. 2A). Provide either a file path to a .tif stack (movie) or a file path to a folder containing single frame .tif files that are labeled in increasing order, e.g. t0001.tif, t0002.tif, t0003.tif.

If one or more movies are already open in Fiji, the startup dialog window offers more choices (Fig. 2B). The user can select to annotate an already opened movie by selecting ‘Already opened movie’ and choosing the movie from a list of already opened movies. If the user decides to open a new movie from a file path, select ‘New movie’ and provide a file path in the ‘Load movie from file path (if ‘New movie’) selected’ field. Provide either a file path to a .tif stack (movie) or a file path to a folder containing single frame .tif files that are labeled in increasing order, e.g. t0001.tif, t0002.tif, t0003.tif.

### Annotation of a movie

**Fig. 3.**
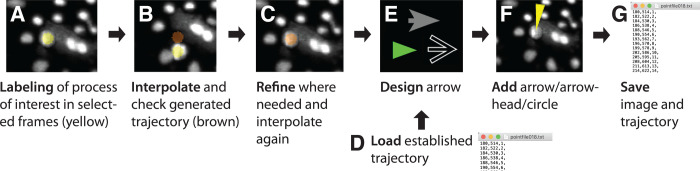
**Workflow of annotation process.** The workflow is comprised of (A) labeling of your process of interest in selected frames, (B) interpolation to establish the trajectory also in frames that were not labeled, (C) iterative refinement of the trajectory until the process of interest is correctly labeled, (D) instead of generating a new trajectory, an already established trajectory can be loaded, (E) design your desired graphical symbol, such as an arrow, arrowhead or circle, (F) adding the arrow to the movie in the proper orientation and distance to the process of interest, and (G) saving the annotated movie and trajectory.
B. After labeling the process of interest in few selected frames, press ‘Interpolate (i)’ in the arrow options panel or ‘i’ on your keyboard. The interpolation first calculates for every frame with labels the median position of all clicked points in the frame. Note: the points are not averaged but the median of the points is taken for the following reason: a median ignores outliers, and therefore clicking several times at the right location easily overrides a miss-click. While this sounds like a cumbersome way to operate the plugin at first, it is easy to click several times at the right location. For example, one miss-click is corrected by two clicks at the right location, two miss-clicks by three clicks. This is also much faster than, for example, manually editing a text file to remove miss-clicks. Overall, taking the median for interpolation ensures that you can ‘override’ easily a label at a wrong position in step A by clicking once again.
  After calculating the median in every frame with labels, the plugin calculates the trajectory between the first and last selected frame of the process of interest by linear interpolation. The newly calculated trajectory is displayed in the movie as brown circles between the first and last selected frames.
C. After the brown interpolated trajectory is displayed, check whether the process of interest is labeled correctly throughout all frames. If this is not the case as the movement is not linear, refine it by repeating step A and B, i.e. click on selected frames and interpolate until you are satisfied with the established trajectory.
D. If you are annotating a new movie, skip this step. If you have already established a trajectory, e.g. for another channel of the movie, load the trajectory by pressing ‘Load points (l)’ in the array option panel or by pressing ‘l’ on the keyboard. This will open a dialog window to specify the file path to an established trajectory. Please provide a .txt file with a list of points. The points in the .txt file should be provided as one line per frame in the format: ‘x-position, y-position, frame number,’. To display the brown labels, press the button ‘Interpolate (i)’ in the arrow options panel or ‘i’ on your keyboard. If you do not want to interpolate, directly proceed to step E and add the graphical symbol of choice. Note that the loading of new points deletes any already selected points.
E. If you are satisfied with the trajectory, customize your desired graphical symbol in the array option panel. There are five different available arrow head styles, an option to fill the shape, different available colors, and parameters to adjust the length of the arrow head, the overall length of the arrow, and its thickness. To preview the arrow as it will be displayed in the movie, an arrow preview window is updated with every change of parameters.
F. To finally add the designed arrow to the movie, press the button ‘Draw Arrows (d)’ or the key ‘d’ on your keyboard. To orient the arrow in an arbitrary angle relative to the position or to assign an x- or y-shift to the arrow position, adjust the parameters ‘Arrow Angle’, ‘Move arrow by x’, ‘Move arrow by y’ in the arrow options panel. To update the graphical symbol in the movie, first remove already drawn symbols by clicking ‘Remove arrows (r)’ in the arrow option panel or press the key ‘r’ on your keyboard. Now, add the new graphical symbol again by pressing the button ‘Draw Arrows (d)’ or the key ‘d’ on your keyboard.
G. Lastly, to save the annotation, add the graphical symbol first permanently to the image by clicking ‘Permanently add overlay’. Now, save the movie in your preferred file format, e.g. Tiff or .avi movie, using the Fiji command File>Save As. Moreover, to reuse the trajectory, save it by pressing the button ‘Save points (s)’ or the key ‘s’ on your keyboard. This will open a dialog window to specify where the points are saved as a text file. The points in the .txt file are saved one line per frame in the format: ‘x-position, y-position, frame number,’.
  Note 1: Pressing the keys on the keyboard only works if the movie is in the foreground, i.e. select it by clicking on it.
  Note 2: The trajectory file can be modified outside of the plugin, e.g. to remove annotations for selected frames. Loading the modified trajectory will not interpolate the points automatically and you could directly add your graphical symbol of choice to the movie.
  Note 3: To restart the annotation process, press ‘Remove arrows (r)’ in the arrow option panel or ‘r’ on the keyboard, and press ‘Remove points’.
  Note 4: If you want to annotate several trajectories, finish the first annotation and permanently add ‘overlay’. Remove points and start with the second trajectory.
  Note 5: You can open a new movie to annotate by pressing ‘Open new movie’ in the arrow option panel. Please note that the current list of points is not automatically deleted by loading a new movie, which allows one to copy an established trajectory to another movie. Alternatively, delete all points by pressing ‘Remove points’ before annotating the new movie.

The workflow to annotate the movie consists of first establishing or loading a trajectory, then selecting and modifying the desired graphical symbol such as an arrow, arrowhead, or circle, and then lastly save the annotated movie and/or trajectory (Fig. 3). In detail, the subsequent steps should be followed:

A. If you want to load an already established trajectory, skip to step D. To annotate a movie for the first time, select the rectangle tool in the Fiji toolbar and click with the mouse on the selected process of interest at its first frame of occurrence in the movie. A yellow circle now labels the position where you clicked. If you miss-click, correct it by clicking few times at the right position (see note in step B). Repeat this for the last frame of occurrence of your process of interest and on selected frames in-between.

## DISCUSSION

We have presented a user-friendly Fiji plugin that simplifies annotation of stationary and non-stationary processes of interest in movies with custom symbols such as arrows, circles or arrowheads. It facilitates this process by providing an intuitive graphical user interface that enables clicking on the process of interest in selected frames to establish a trajectory. Moreover, the layout and buttons of the option panel intuitively guide the user through the annotation process.

Importantly, to reduce the number of frames that have to be labeled, the plugin interpolates between frames. This feature relies on linear interpolation that is most powerful when the process of interest moves linearly, i.e. with a constant velocity. This includes stationary processes as their velocity is constantly zero or processes such as cell migration or flows at constant velocities. On the other hand, processes that evolve in more complex ways, such as on random or zigzag trajectories, require a manually inserted label at every frame where the velocity changes, reducing the effectiveness of linear interpolation. To completely automate the establishment of trajectories of such complex motions, more complicated algorithms are devisable. However, the time needed to compute these trajectories reduces their appeal in a simple-to-use plugin. Therefore, we decided to only offer linear interpolation in the plugin. Nevertheless, the plugin is capable to import established trajectories from other software packages over its easy to use import functionality.

In conclusion, we envision that our tool is particularly useful for annotating long-term time-lapse movies highlighting processes such as specific developmental programs, rare cell behaviors in cancer or immune cell interactions. The established trajectories might also be a starting point for the generation of training data for automated identification of such processes by deep learning algorithms and other neuronal networks.

## MATERIALS AND METHODS

The Fiji plugin was written in Java as an ImageJ 1.x plugin and is available on github https://github.com/DaetwylerStephan/draw_arrow_in_movies or in the Supplementary Material of this paper. It consists of four classes, which are described below.

### ‘SPIM_DrawArrowInMovie_ .java’ class

The ‘SPIM_DrawArrowInMovie_ .java’ is the main plugin class. In the run method, first the method ‘openMovie()’ is called. This method first loads and displays the movie as ImagePlus object. After that it sets the KeyListener and MouseListener to interact with the opened movie. The MouseListener defines how after clicking on a point in a movie frame (mousePressed(MouseEvent e)), (i) the selected point is added to a hashmap ‘pointmap’ that collects all selected points and (ii) a yellow circle at the selected point is added to the overlaytotal that displays all selected points. The KeyListener defines which methods are called by pressing different keys. Pressing ‘i’ calls the ‘interpolate_values()’ method. This method uses the hashmap of all clicked points (‘pointmap’) to calculate an array of interpolated values covering all frames from the first frame to the last frame where points were selected. After interpolation, the overlaytotal is updated to only display the interpolated points as brown circles. Pressing ‘d’ draws the circle, arrow or arrowhead as selected by the user in the option panel implemented in the class ‘ArrowOptionPanel2.java’ as part of the ‘overlayarrow’ overlay on the movie. Pressing ‘s’ calls the ‘save_points()’ method that first opens a GenericDialogPlus window to specify the file path, and then writes all the interpolated values as a text file (‘writepointfile()’). Pressing ‘l’ calls the ‘loadpointfile()’ method that opens a GenericDialogPlus window to specify the file path to a text file with points, and then loads these points as interpolated values and adds them as points to the hashmap ‘pointmap’. Pressing ‘r’ calls the ‘deleteoverlay()’ method that removes all already drawn graphical symbols from the overlayarrow. The ‘SPIM_DrawArrowInMovie_ .java’ further implements the ‘deletepoints()’ method that removes all points from the hashmap ‘pointmap’ and the overlay ‘overlaytotal’, and the ‘flattenimage()’ method that permanently adds the current overlay to the image by flattening the ImagePlus object. These two last methods are called from pressing a JButton object in the ‘ArrowOptionPanel2.java’ class.

### ‘ArrowOptionPanel2.java’ class

The ‘ArrowOptionPanel2.java’ class extends the ‘ArrowOptionPanel.java’ from the Arrow Tool by Jean-Yves Tinevez (Tinevez et al., 2017). It provides a javax.swing.JFrame graphical user interface where the user can manually define the parameters of the arrow shape and how the arrow is positioned in regard to the selected points. It further implements buttons as JButtons to call all methods of the ‘SPIM_DrawArrowInMovie_ .java’ class for opening a new movie, interpolation, labeling, loading and saving the labels. In addition, a help/instruction window can be opened by pressing a JButton.

### ‘ArrowShapeV2.java’ class

The ‘ArrowShapeV2.java’ class defines five different shapes (DELTA, THICK, THIN, CIRCLE, POINTY) for an arrow, arrowhead or circle. It extends the class ‘ArrowShape.java’ by Jean-Yves Tinevez (Tinevez et al., 2017).

### ‘InterpolateHashMap.java’ class

The class ‘InterpolateHashMap.java’ is called in the ‘SPIM_DrawArrowInMovie_ .java’ class from the method ‘interpolate_values()’ after pressing the letter ‘i’ in the image or pressing the JButton ‘Interpolate (i)’ in the ‘ArrowOptionPanel2.java’ class. It takes the hashmap ‘pointmap’ of selected points from the movie and generates an array of linearly interpolated values that approximate the trajectory of the process of interest.

## Supplementary Material

Supplementary information
