# Supplementary material for: Fiji plugin for annotating movies with custom arrows
Source: Biol Open. 2020 Dec 1;9(11):bio056200. doi: 10.1242/bio.056200 (PMC7725597; doi:10.1242/bio.056200)
Supplement: Supplementary information [file biolopen-9-056200-s1.pdf]

Supplementary Code

Click here to Download Supplementary Code

Biology Open • Supplementary information
